# Inspiratory muscle training in weaning from prolonged mechanical ventilation: a systematic review and meta-analysis

**DOI:** 10.3389/fmed.2025.1719837

**Published:** 2026-01-13

**Authors:** Francisca A. Andrade-Rebolledo, Guillermo Villagra-Morales, Leonardo A. Pérez

**Affiliations:** 1 Escuela de Kinesiología, Facultad de Medicina y Ciencias de la Salud, Universidad Mayor, Santiago, Chile; 2 Escuela de Kinesiología, Facultad de Ciencias de la Rehabilitación y Calidad de Vida, Universidad San Sebastián, Santiago, Chile

**Keywords:** inspiratory muscle training, intensive care units, prolonged mechanical ventilation, respiratory muscle training, weaning

## Abstract

Prolonged mechanical ventilation in intensive care unit (ICU) patients poses significant challenges, including increased morbidity and healthcare costs. Effective weaning strategies are critical to improving patient's outcomes, yet the optimal approach remains unclear. Inspiratory muscle training (IMT) has emerged as a potential adjunct therapy to facilitate weaning, addressing the gap in standardized interventions. This systematic review and meta-analysis evaluate the effectiveness of IMT on weaning from mechanical ventilation in ICU patients. Seven randomized controlled trials published since 2019 were analyzed, comparing various IMT protocols. The findings support that high-intensity IMT can enhance extubation success rates and reduce the duration of mechanical ventilation. However, considerable variability in protocols and patient populations limits the generalizability of these findings. The findings underscore the urgent need for further research to standardize IMT protocols and to identify the patient subgroups most likely to benefit. Future studies should focus on refining methodologies and developing consensus-based guidelines to maximize the effectiveness of IMT in ICU settings. This review highlights the potential of IMT as a targeted strategy to improve weaning outcomes, reinforcing the importance of advancing evidence-based practices in critical care.

**Systematic review registration:**

https://www.crd.york.ac.uk/prospero/, identifier: CRD420251070529.

## 1 Introduction

During the past decade, mortality associated with critical illnesses has decreased globally (1), highlighting an increased demand for effective rehabilitation strategies. The advanced management of ventilatory support, along with the increase in severe cases such as ARDS, has led to more patients with prolonged mechanical ventilation dependency and the need for post-extubation pulmonary rehabilitation (2).

**Mechanical ventilation (MV)** is widely used as a therapeutic support for respiratory function, enhancing adequate gas exchange and reducing respiratory effort (3, 4). However, prolonged use of MV may lead to adverse effects, particularly on the diaphragm muscle, through a mechanism of myotrauma that results in **ventilator-induced diaphragm dysfunction** [VIDD; (5, 6)]. VIDD is a prevalent phenomenon characterized by the progressive loss of diaphragmatic muscle strength due to MV (7). VIDD can occur within 18–48 h of mechanical ventilation, contributing to difficulties in the weaning process and affecting up to 80% of patients (8–11). It is also associated with poor outcomes in critically ill patients (12–20). This dysfunction is linked to oxidative stress, alterations in proteolytic pathways, and mitochondrial dysfunction, resulting in muscle atrophy, strength loss, and reduced diaphragmatic mobility (21, 22).

One strategy to facilitate weaning from MV is Respiratory Muscle Training (RMT), including specific modalities such as Inspiratory Muscle Training [IMT; (23–26)]. RMT is frequently employed in intensive care units (ICU) as a low-cost and easy-to-implement intervention designed to enhance the strength and endurance of ventilatory muscles, notably the diaphragm and intercostal muscles (27–29). This training is typically performed using devices that impose resistance to airflow during inspiration or expiration, generating a controlled overload that induces physiological adaptations aimed at improving respiratory efficiency (28, 29).

Inspiratory muscle training (IMT) is commonly performed using resistive loading or threshold loading devices, both of which increase the work of breathing to strengthen inspiratory muscles such as diaphragm and intercostals. Resistive loading typically uses an orifice or flow-dependent resistance, so that the load varies with inspiratory flow and volume, eliciting predominantly dynamic (isotonic) contractions characterized by muscle shortening and lengthening against resistance during inspiration. These isotonic contractions enhance inspiratory muscle endurance and strength through repeated dynamic work across a larger range of motion, which can improve maximal inspiratory pressure (MIP) and fatigue resistance (23, 30). Threshold loading, by contrast, employs a spring-loaded or valve-based mechanism that remains closed until a preset inspiratory pressure is generated, thereby imposing a constant pressure load that is largely independent of flow once the threshold is reached. This configuration predominantly elicits isometric contractions, in which inspiratory muscles develop high tension with relatively little change in length, like holding a fixed load, and thereby targets maximal strength and high-threshold motor unit recruitment. Repeated threshold loading has been shown to markedly increase maximal inspiratory pressure and improve both strength and endurance of the inspiratory muscles, which translates into better exercise tolerance and reduced dyspnea in clinical populations such as patients with COPD (24–26).

Several studies have reported that RMT enhances inspiratory muscle strength and endurance, shortens the duration of weaning, and increases the success rate of extubation (31–33). Nonetheless, the overall evidence remains inconclusive, as other investigations have not demonstrated significant differences in weaning time or patient survival (34–37). This discrepancy in findings may be attributed to substantial heterogeneity in study designs and the wide variability in RMT protocols—particularly in terms of training frequency, intensity, duration, and modality (38, 39).

Given these inconsistencies, there is a pressing need for further high-quality research to standardize RMT procedures. Establishing clear, evidence-based guidelines would help optimize its clinical utility, especially in patients requiring prolonged mechanical ventilation (24, 36, 40).

Despite growing evidence for RMT, no systematic review has targeted patients with prolonged mechanical ventilation (>48 h), where ventilatory dysfunction is prevalent and weaning challenges are acute. This gap is critical, as a focused review would directly address muscle weakness, optimize RMT protocols, and establish standardized guidelines to boost efficacy. Such standardization promises to shorten ventilator dependency, reduce ICU length of stay, and lower mortality risk in this high-risk population.

## 2 Methodology

### 2.1 Design

The systematic review and meta-analyses were conducted in accordance with the Preferred Reporting Items for Systematic Reviews and Meta-Analyses (PRISMA) guidelines (27) PROSPERO database was consulted to check the existence of similar systematic reviews. The meta-analysis protocol was also registered in PROSPERO (CRD420251070529).

### 2.2 Search strategy

An exhaustive search was conducted across multiple electronic databases to identify relevant studies on IMT in ICU patients under prolonged mechanical ventilation and undergoing weaning. The selected databases included ScienceDirect, Web of Science, PubMed and Google Scholar. The search was supplemented with a review of references from included articles to ensure no potentially relevant studies were overlooked.

The search strategy was developed using specific terms addressing the topic of interest. The main search terms included: Inspiratory Muscle Training (“Inspiratory Muscle Training” OR “IMT”); Respiratory Muscle Training (“Respiratory Muscle Training” OR “RMT”); Mechanical Ventilation Weaning (“Mechanical Ventilation Weaning” OR “Weaning from Ventilation”); Intensive Care (“Intensive Care Unit” OR “ICU”); Prolonged Ventilation (“Prolonged Mechanical Ventilation” OR “Prolonged Ventilation”). These terms were combined using the Boolean operators “AND,” “OR,” and “NOT.” The exact search strategy was adapted to each database to meet its specific requirements. The search was restricted to studies published from 2019 onwards and in English or Spanish. A total of 377 results were obtained.

### 2.3 Inclusion criteria

The following inclusion criteria will be applied to the studies: (a) Studies involving adult patients (≥18 years) in the Intensive Care Unit (ICU) or Critical Care Unit (CCU) who have received at least 48 h of uninterrupted invasive mechanical ventilation and are currently undergoing the weaning process. This 48-h threshold aligns with established clinical guidelines for identifying prolonged mechanical ventilation, a condition associated with heightened ventilatory muscle dysfunction, weaning challenges, and increased risk of extended ICU dependency. (b) Studies evaluating at least one inspiratory muscle training (IMT) protocol, with no restrictions on frequency, duration, or the type of device used for IMT. (c) Studies comparing different IMT protocols or evaluating an IMT protocol against a control group receiving standard care without IMT intervention or a placebo treatment. (d) Studies that assess outcomes such as successful weaning rates, total duration of mechanical ventilation, changes in maximal inspiratory pressure (MIP), and ICU or CCU mortality. (e) Randomized controlled trials (RCTs) assessing the effects of different IMT protocols will be included.

### 2.4 Exclusion criteria

Studies meeting any of the following criteria will be excluded: (a) Studies published in languages other than English or Spanish, unless an official translation is available. (b) Studies involving patients in pre- or postoperative periods. (c) Studies including patients diagnosed with heart failure^*^. (d) Studies involving oncology patients or those with active cancer^**^. (e) Studies including pregnant or postpartum patients. (f) Studies involving non-cooperative patients or those with cognitive impairments prevent active participation in IMT. (g) Studies with pediatric or neonatal participants (under 18 years old). (h) Studies that do not detail or specifically describe the implemented IMT protocol. ^*^Patients with heart failure exhibit significant alterations in hemodynamic and respiratory functions, which can negatively impact the outcomes of respiratory muscle training (RMT). These alterations include increased left ventricular diastolic pressure, chronic hyperventilation and hypoxia, and generalized muscle weakness. which may compromise the validity and generalizability of study results (28, 29). ^**^Studies involving oncology patients or those with active cancer were excluded due to their unique physiological and clinical characteristics. Cancer-related cachexia, treatment-induced pulmonary toxicity, and immunosuppression significantly impact respiratory muscle function and training outcomes. Moreover, the heterogeneity in disease stage, therapeutic approaches, and associated complications, such as infections and thromboembolism, limits the generalizability of findings to other critically ill population (31, 32).

### 2.5 Data analysis

The Rayyan Pro™ platform was utilized for managing the study selection process, while statistical analyses was performed using Python. Mean and Standard Deviation were used to compare the treatment protocols while all effect sizes are presented with their corresponding 95% confidence intervals (CI). Quantitative analysis was conducted using the inverse variance (IV) method. A random-effects model was applied in all analyses to estimate the overall effect size, accounting for the limited number of included studies. Statistical significance was defined as *p* < 0.00001.

### 2.6 Risk of bias

The RoB 2 tool (33) was selected and used to assess five key domains (risk of bias arising from the randomization process, risk of bias due to deviations from the intended interventions, risk of bias due to missing outcome data, risk of bias in measurement of the outcome, and risk of bias in selection of the reported result), classifying the bias as “low risk of bias”, “some concerns” and “high risk of bias”.

## 3 Results

### 3.1 Study selection

**Figure 1:**
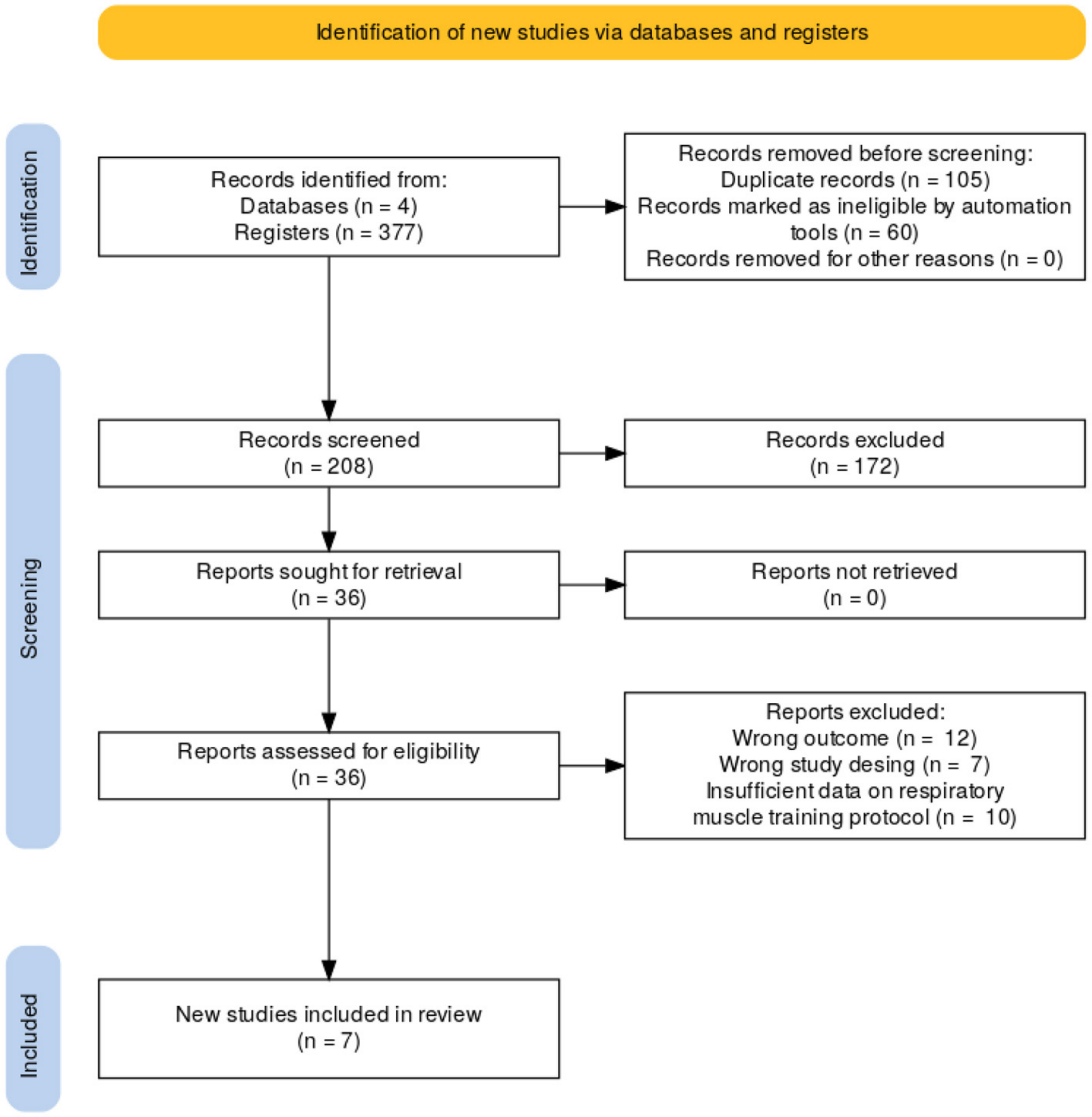
Flow diagram of the literature search.

The study selection process is shown in Figure 1, where the database search retrieved 377 studies and it was conducted in several stages, first, **Duplicate Removal**: After gathering records from all databases, duplicate articles were removed, resulting in a total of 268 studies for review. Second, **Title and Abstract Screening**: The titles and abstracts of the articles were reviewed, excluding those that did not meet the predefined inclusion criteria. At this stage, 162 studies were excluded for being unrelated to the topic or failing to meet inclusion criteria. Then, **Full-Text Evaluation**: The full texts of the remaining 36 studies were reviewed to assess their eligibility based on inclusion and exclusion criteria. During this phase, 29 articles were excluded for reasons such as inadequate outcomes, non-randomized design, or lack of specific data on the training protocol. Finally, **Included Articles**: 7 studies met all inclusion criteria and were considered of high methodological quality to address the research question posed in this review.

### 3.2 Bias analysis

**Figure 2:**
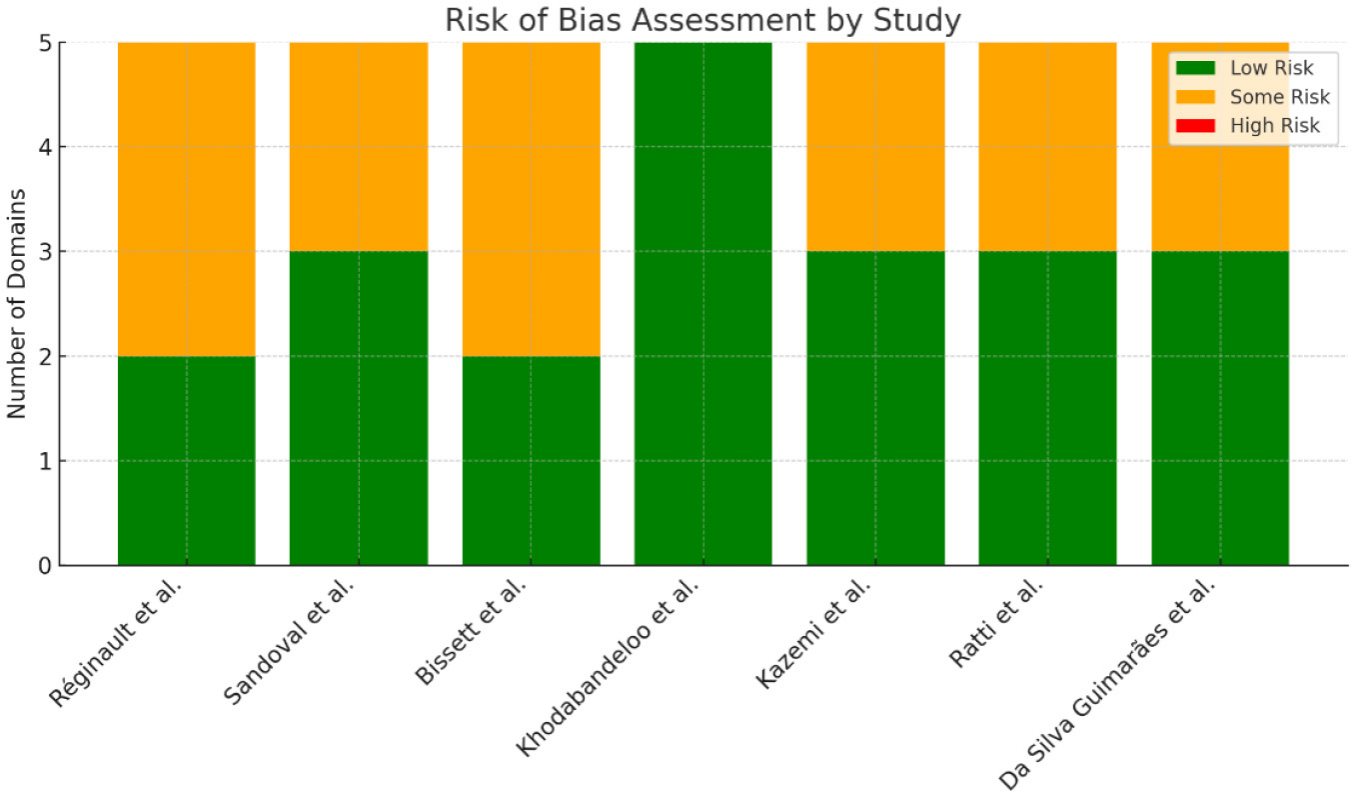
Risk of bias analysis.

We conducted a risk of bias analysis on the selected studies, revealing that the majority exhibited some level of bias. This was primarily attributed to the absence of blinding for both participants and interventions (Figure 2). Each item was classified as low (green), unclear (yellow) or high (red) risk of bias.

### 3.3 Interventions

A total of 642 ICU patients were included among different training strategies. All results are comprehensively summarized in Table 1. Notably, studies such as those by da Silva et al. (36), Khodabandeloo et al. (34), and Kazemi et al. (35) demonstrated significant improvements in MIP, higher rates of successful weaning, and reduced duration of mechanical ventilation (34–36). For instance, Khodabandeloo et al. utilized a high-frequency protocol with progressive loading, achieving notable gains in respiratory strength and independence. Similarly, Kazemi et al. (35) highlighted improvements not only in MIP but also in diaphragm thickness and mobility, further supporting the physiological benefits of high-intensity IMT. In contrast, studies employing lower-intensity or shorter-duration protocols, such as Réginault et al. (38) and Sandoval Moreno et al. (37), did not yield significant results, likely due to insufficient stimulus or inadequate timeframes for adaptation.

**Table 1:** Characteristics of included studies.

**Author, year**	**Sample size**	**Intervention**	**Comparison**	**Study conclusion**
Bissett et al., 2023 (39)	70 (33 IMT, 37 control)	Once a day, 5 times per week for two weeks. Intensity at 30–50% of the MIP	Control group with standard care, no IMT	IMT is useful for improving quality of life and dyspnea but has no impact on MIP
Réginault et al., 2024 (38)	92	Three IMT protocols (low, high, and mixed intensity), 2 times/day, 7 days/week, until successful extubation or 30 days	Comparison among three intensities of EMR, without a control group	No significant benefits in strength or endurance were observed with any modality
Sandoval Moreno et al., 2019 (37)	126	IMT with a threshold device at 50% of MIP, 3 sets of 6–10 breaths, twice a day	Standard care with respiratory physiotherapy	IMT did not show efficacy in reducing weaning time or improving muscle strength in this population
Guimarães et al., 2021 (36)	101	IMT with an electronic resistive device, 2 sets of 30 breaths each, with an initial load set at 40% of the MIP, progressively increasing until the target load	Control group with T-piece trial without IMT	Significant improvement in weaning success (74.8% vs. 44.5%) and 60-day survival in the IMT group.
Khodabandeloo et al., 2023 (34)	79	Inspiratory muscle training with a threshold device (50% of MIP, increasing to tolerance), five sets of six breaths per day	Standard care with conventional physiotherapy	IMT group significantly reduced weaning time and improved MIP and RSBI
Ratti et al. 2022 (40)	104	Two daily sessions (morning and afternoon), 7 days a week. Each session consisted of 30 breaths divided into 3 sets of 10 breaths, with 1-min rest intervals between sets	Spontaneous breathing with T-piece	Different types of IMT showed no significant benefits on weaning
Kazemi et al. 2024 (35)	70	5 sets of 10 breaths twice a day for one week	Standard respiratory care	The combination of IMT and PEP with a threshold device improves pulmonary function and extubation success in ICU patients

Some studies revealed key limitations that influence the interpretation of results. For example, Bissett et al. (39) employed a two-week protocol with a small sample size, which may have been insufficient to produce sustainable improvements in MIP. Similarly, Réginault et al. (38) compared different IMT intensities, potentially diluting the impact of individual protocol. While Da Silva et al. demonstrated positive outcomes (36), their single-center design and lack of long-term follow-up limit the generalizability of their findings. Roceto Ratti et al. (40), despite including a relatively large sample (*n* = 132), encountered challenges related to the state of wakefulness, with a Glasgow Coma Scale score below 8, complicating active participation in training.

Device selection also played a critical role in outcomes. Studies using electronic devices like POWERbreathe™, as seen in da Silva Guimarães et al. (36) and Roceto Ratti et al. (40), often achieved better results compared to threshold devices. However, the feasibility of such devices in resource-limited ICU settings remains a challenge. Additionally, findings from Bissett et al. (39) suggest that even when significant improvements in MIP or MV duration are not observed, IMT can positively impact patients' quality of life, emphasizing the broader benefits of such interventions.

The heterogeneity in study designs, protocol parameters, and patient characteristics remains a significant barrier to generalizing results. Key factors such as intensity, frequency, and total training duration were found to influence outcomes. For instance, longer-duration protocols like Da Silva et al. (36) (60 days) tended to report superior results compared to shorter interventions like those by Réginault et al. (38) (7 days). Moreover, studies with progressive loading and higher respiratory volumes, such as Khodabandeloo et al. (34), consistently outperformed those with fixed, lower-intensity training regimens.

**Figure 3:**
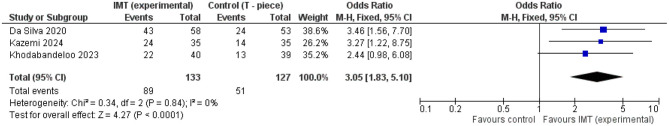
Weaning success.

The forest plot (Figure 3) shows that overall RMT protocols have a positive impact on weaning success. da Silva et al. (36) appear to be the most influential study, possibly due to the use of electronic devices or longer protocols. Studies with wide CIs or results below the combined average, such as Sandoval et al. (37), suggest that factors such as intensity or duration may have negatively influenced weaning. Some studies, including those by da Silva, Khodabandeloo, and Kazemi, demonstrated that IMT can enhance weaning success rates (34–36). In contrast, studies such as those conducted by Bissett, Roceto Ratti, Sandoval, and Réginault et al., failed to demonstrate significant differences in increasing weaning success (38–40). These inconclusive results may be attributed to factors such as limited sample size, variability in the IMT protocols (Table 2), and population heterogeneity.

**Table 2:** Training variables.

**Study**	**Frequency (*F*)**	**Intensity (*I*)**	**Volume of training**	**Total program duration**	**Session duration**	**Type (*T*)**
Réginault et al. (38)	Twice daily, 7 days/week	HI: against maximal load tolerated	HI: 4 sets of 6 breath.	Until extubation or max. 30 days	Approximately 14.5 ± 5.2 min, including a 5-min post-training recovery period	Threshold IMT
		LI: 5 min against a load of 30% initial MIP with 10% increase per day	LI: 5 min			
		MI: against a incremental load from 30% to 60% of daily's MIP	MI: 4 sets of 20 breaths			
Sandoval Moreno et al. (37)	Twice daily	50% of MIP, adjusted daily with rest intervals	3 sets of 6–10 breaths	Approximately 20 min per session	Until the patients were successfully weaned from mechanical ventilation or experienced weaning failure	Threshold IMT
Bissett et al. (39)	Once daily	High intensity (≥50% of MIP); 30 breaths per session	5 sets of 6 breaths	Around 15 min per session	From the start of randomization until 1 week after successful liberation from mechanical ventilation	Threshold IMT
Khodabandeloo et al. (34)	Once daily	Starts at 50% of MIP, progressively increased	5 sets of 6 repetitions each	Varies per session; monitored weekly	Until successful weaning or failure	Threshold IMT
Kazemi et al. (35)	Twice daily, 1 week	50% of MIP, increasing de load to tolerance	5 sets of 10 breaths	Roughly 15–20 min per session	7 days or until extubation was achieved	Threshold IMT + PEP exercises
Roceto Ratti et al. (40)	Twice daily	Manual: load set at 30% of maximal inspiratory pressure (MIP), with daily increments of 10% based on tolerance	3 sets of 10 breaths	About 15 min per session	Until the participants were successfully weaned from mechanical ventilation or until the decision to discontinue the weaning process was made	Electronically assisted IMT or T-piece breathing
		Automatic: Load: the device automatically adjusted the load based on the maximal effort exerted by the patient during the first two breaths of each session				
Da Silva Guimarães et al. (42)	Once daily	30–50% of MIP, adjusted weekly	3 sets of 10 breaths	20–30 min per session	Until successful weaning	Electronic resistive loading device (POWERbreathe K-5™)

The asterisk (^*^) indicates training intensity classification: HI, high intensity; LI, low intensity; MI, moderate intensity.

**Figure 4:**
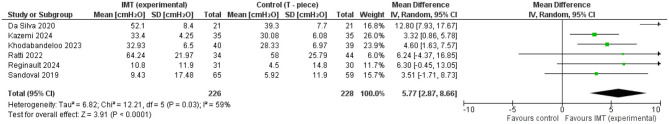
Changes in MIP (maximal inspiratory pressure). **High-intensity inspiratory muscle training (IMT)** is associated with a **significant improvement in MIP** compared to low-intensity IMT or usual care, with a pooled mean difference of **6.13 cm H**_**2**_**O** (95% *CI*: 3.15–9.11), and **moderate heterogeneity (*I***^2^ **=** **59%)**. Among the six included studies, four showed statistically significant differences favoring high-intensity IMT, while two had confidence intervals crossing zero and were not statistically significant. The overall effect was statistically significant.

Regarding the MIP the forest plot clearly illustrates the significant changes (Figure 4). da Silva Guimarães et al. (36) demonstrated the greatest impact, with an average improvement of 18.6 cm H_2_O in the intervention group. Khodabandeloo et al. (34) and Kazemi et al. (35) also consistently showed statistically significant benefits. Non-significant studies studies, such as Réginault et al. (38), may have been limited by small sample sizes, low-intensity protocols, or participant variability.

## 4 Discussion

IMT has been extensively studied in the ICU as a strategy to enhance weaning capacity and reduce dependence on mechanical ventilation in critically ill patients. This systematic review and meta-analysis evaluated seven randomized studies comparing various IMT protocols in patients with prolonged MV, focusing on their effects on weaning outcomes.

### 4.1 Weaning success rate and duration of mechanical ventilation

The review indicates that high-intensity IMT protocols have a positive impact on weaning success (Table 3), particularly in patients with low baseline Maximal Inspiratory Pressure (MIP) or inspiratory muscle weakness. da Silva Guimarães et al. (36), Khodabandeloo et al. (34) and Kazemi et al. (35) reported significant improvements in successful extubation and reductions in MV duration compared to control groups. These findings suggest that IMT with high training loads (≥50% MIP) enhances inspiratory muscle strength and endurance, which are critical for independent breathing. However, other studies such as Réginault et al. (38) and Roceto Ratti et al. (40), did not find significant differences in outcomes when lower-intensity protocols (< 40% MIP) or less frequency were used. Variations in outcomes may be attributed to differences in the frequency and duration of IMT sessions, as well as baseline patient characteristics.

**Table 3:** Successful weaning rate.

**Study**	**Type of intervention**	***N* (intervention)**	***N* (total)**	**Successful weaning in intervention (%)**	**Successful weaning in control (%)**	**ρ value**	**IC (95%)**
Da Silva et al.	Electronic device (POWERbreathe K-5™)	48	101	74.8%	44.5%	ρ < 0.001	[62.5%, 87,1%]
Ratti et al.	Electronic device	18	104	88%	88%	ρ < 0.05	[73%, 100%]
Ratti et al.	Manual device	16	104	75%	88%	ρ < 0.05	[54%, 96%]
Sandoval et al.	Threshold IMT	62	126	75.81%	75%	ρ > 0.54	[65%, 86%]^*^
Bissett et al.	Threshold IMT	33	70	54.55%	24.32%	ρ > 0.05	[38%, 71%]^*^
Khodabandeloo et al.	Threhold IMT	40	79	55%	33.3%	ρ = 0.042	[45%, 65%]^*^
Kazemi et al.	Threshold IMT	35	70	74.3%	48.6%	ρ = 0.03	[64%, 84%]
Reginault MI	Threshold IMT	43	92	83.7%	Not applicable	ρ = 0.69	[69.3%, 93.2%]
Reginault HI	Threshold IMT	23	92	82.6%	Not applicable	ρ = 0.6091	[61.2%, 95.0%]
Reginault LI	|Threshold IMT	23	92	73.9%	Not applicable	ρ = 0.609	[51.6%, 89.8%]

The asterisk (^*^) indicates studies with non-statistically significant differences or confidence intervals overlapping between groups.

### 4.2 Increase in inspiratory muscle strength and other respiratory parameters

MIP is a key indicator of IMT effectiveness. Some studies reported significant MIP improvements in IMT groups (34, 37), suggesting that training can counteract diaphragmatic atrophy and restore muscle function. Otherwise, studies employing lower intensity or shorter-duration protocols, such as Réginault et al. and Sandoval Moreno et al., did not achieve significant results, underscoring the importance of high intensity in IMT (38, 40). Kazemi et al. (35) also highlighted increased diaphragm thickness and mobility, which may be crucial for functional recovery in the ICU.

### 4.3 Quality of life and ICU survival

Some studies assessed quality of life and survival as secondary outcomes. Bissett et al. (39) assessed quality of life using the SF-36v2 and EQ-5D-3L questionnaires, both validated for critically ill patients. The SF-36v2 evaluates specific domains such as physical function, mental health, and vitality, while the EQ-5D-3L measures general health perception and quality of life. The IMT group showed significant improvements in the physical component score of the SF-36v2 (+6.4 points, 95% CI: 1.96–12.00) and the visual analog scale of the EQ-5D (+17.2 points, 95% CI: 1.3–33.0), although significant changes in MIP or MV duration were not observed (39). This suggests that while IMT may enhance the overall patient experience, its impact on other key physiological outcomes remains uncertain. On the other hand, da Silva Guimarães et al. (36) found higher 60-day survival rates in the IMT group, indicating that training intensity and device type may influence long-term benefits. However, these findings require further validation through multicentric studies with extended follow-up periods.

### 4.4 Limitations of the included studies

The duration of some IMT protocols was often insufficient to capture sustainable respiratory function improvements (37, 38). Participant heterogeneity and differences in patient health status impacted study comparability, with varying definitions of prolonged mechanical ventilation (e.g., >48 h vs. >7 days) emerging as the primary factor influencing overall effect sizes. Studies using shorter thresholds (≥48 h) often reported larger effects on weaning success and inspiratory muscle strength due to earlier intervention timing, capturing patients at higher risk of diaphragmatic atrophy during the acute phase. In contrast, those with longer cutoffs diluted effects by including later-stage patients with entrenched muscle dysfunction, underscoring the need for standardized definitions to enhance meta-analytic precision and clinical applicability. Variability in the devices used and the lack of standardized frequency and intensity of interventions further complicated the synthesis of findings. On the other hand, none of included studies reported the type of feeding received by patients, this topic has been reported as determinant cue to successful weaning in ICU patients (41) and should be addressed in futures studies.

### 4.5 Clinical implications and recommendations

The analysis suggests that intensities above 50% of IMT may be an effective strategy to improve weaning success and reduce MV duration. However, the lack of protocol standardization limits the widespread implementation of these practices. Future studies should focus on creating uniform protocols that optimize training load, frequency, and device type. Regarding electronic devices, the evidence is not consistent enough to claim they are superior to other types of threshold devices, as only da Silva Guimarães et al. (36) reported positive effects on MIP and weaning. Therefore, it would be more appropriate to focus on the intensity and frequency of IMT as key factors in the effectiveness of the training.

### 4.6 Future directions

It is essential to conduct multicentric trials with larger, more diverse samples to validate these findings and assess the long-term benefits of IMT. Research should also explore the combination of IMT with other interventions, such as positive expiratory pressure (PEP), to maximize respiratory benefits.

## 5 Conclusion

Inspiratory muscle training (IMT) plays a pivotal role in facilitating weaning from prolonged mechanical ventilation in critically ill patients. Evidence consistently demonstrates that IMT enhances inspiratory muscle strength, endurance, and diaphragmatic function, key components for successful weaning. Notably, the effectiveness of IMT is closely tied to the protocol employed. High-intensity protocols (≥60% of maximal inspiratory pressure) are associated with better outcomes, including increased weaning success rates and shorter durations of mechanical ventilation. However, despite its promise, IMT implementation remains hindered by protocol variability, lack of standardization, and inconsistencies in study design. Future research should focus on identifying optimal IMT practices and their applicability across diverse ICU settings to promote consistent and evidence-based outcomes.

## Data Availability

The original contributions presented in the study are included in the article/supplementary material, further inquiries can be directed to the corresponding author/s.
