# Supplementary material for: Inspiratory muscle training in weaning from prolonged mechanical ventilation: a systematic review and meta-analysis
Source: Front Med (Lausanne). 2026 Jan 13;12:1719837. doi: 10.3389/fmed.2025.1719837 (PMC12835378; doi:10.3389/fmed.2025.1719837)
Supplement: Supplementary file 1 [file Table_1.docx]

Supplementary Material

# Mesh Terms

The list of Mesh terms used as complement to primary search terms and Boolean operators.

- Breathing Exercises
- Respiration, Artificial
- Ventilator Weaning
- Intensive Care Units

# Search strategies adapted by database

| **Database** | **Search Strategy** |
| --- | --- |
| ScienceDirect | {Inspiratory Muscle Training AND Mechanical Ventilation Weaning AND Prolonged Mechanical Ventilation AND Intensive Care} or {Respiratory Muscle Training AND Weaning from Ventilation AND Intensive Care Unit} or {Inspiratory Muscle Training OR Respiratory Muscle Training AND Mechanical Ventilation Weaning AND Prolonged Mechanical Ventilation} |
| Web of Science | {Inspiratory Muscle Training AND Mechanical Ventilation Weaning AND Prolonged Mechanical Ventilation AND Intensive Care} or {Respiratory Muscle Training AND Weaning from Ventilation AND Intensive Care Unit} or {Inspiratory Muscle Training AND Mechanical Ventilation Weaning OR Weaning from Ventilation AND Prolonged Mechanical Ventilation AND Intensive Care OR ICU} |
| PubMed | {Inspiratory Muscle Training AND Mechanical Ventilation Weaning AND Prolonged Mechanical Ventilation AND Intensive Care} or {Respiratory Muscle Training AND Weaning from Ventilation AND Intensive Care Unit} or {Breathing Exercises AND Prolonged Mechanical Ventilation AND Weaning from Ventilation} |
| Google Scholar | {Inspiratory Muscle Training AND Mechanical Ventilation Weaning AND Prolonged Mechanical Ventilation AND Intensive Care} or {Respiratory Muscle Training AND Weaning from Ventilation AND Intensive Care Unit} or {Inspiratory Muscle Training AND Mechanical Ventilation Weaning OR Weaning from Ventilation AND Prolonged Mechanical Ventilation AND Intensive Care OR ICU} or {Breathing Exercises AND Ventilator Weaning OR Weaning from Ventilation AND Prolonged Mechanical Ventilation AND Intensive Care OR ICU} |
